# Modifiable risk factors for cancer among people with lynch syndrome: an international, cross-sectional survey

**DOI:** 10.1186/s13053-024-00280-w

**Published:** 2024-06-14

**Authors:** Robert F. Power, Damien E. Doherty, Roberta Horgan, Pat Fahey, David J. Gallagher, Maeve A. Lowery, Karen A. Cadoo

**Affiliations:** 1 School of Medicine, Trinity College Dublin, Dublin, Ireland; 2 Cancer Genetics service, Trinity St James’s Cancer Institute, Dublin, Ireland; 3 Mater Misericordiae University Hospital, Eccles Street, Dublin, Ireland; 4 Lynch syndrome Ireland, Dublin, Ireland; 5 Department of Medical Oncology, Trinity St James’s Cancer Institute, Dublin, Ireland

**Keywords:** Lynch syndrome, Colorectal cancer, Endometrial cancer, Modifiable risk factors, Cancer prevention

## Abstract

**Background:**

Lynch syndrome is the most common cause of hereditary colorectal and endometrial cancer. Lifestyle modification may provide an opportunity for adjunctive cancer prevention. In this study, we aimed to characterise modifiable risk factors in people with Lynch syndrome and compare this with international guidelines for cancer prevention.

**Methods:**

A cross-sectional study was carried out utilizing survey methodology. Following public and patient involvement, the survey was disseminated through patient advocacy groups and by social media. Self-reported demographic and health behaviours were collected in April 2023. Guidelines from the World Cancer Research Fund (WCRF) were used to compare percentage adherence to 9 lifestyle recommendations, including diet, physical activity, weight, and alcohol intake. Median adherence scores, as a surrogate for lifestyle risk, were calculated and compared between groups.

**Results:**

156 individuals with Lynch syndrome participated from 13 countries. The median age was 51, and 54% were cancer survivors. The mean BMI was 26.7 and the mean weekly duration of moderate to vigorous physical activity was 90 min. Median weekly consumption of ethanol was 60 g, and 3% reported current smoking. Adherence to WCRF recommendations for cancer prevention ranged from 9 to 73%, with all but one recommendation having < 50% adherence. The median adherence score was 2.5 out of 7. There was no significant association between median adherence scores and age (*p* = 0.27), sex (*p* = 0.31), or cancer history (*p* = 0.75).

**Conclusions:**

We have characterised the modifiable risk profile of people living with Lynch syndrome, outlining targets for intervention based on lifestyle guidelines for the general population. As evidence supporting the relevance of modifiable factors in Lynch syndrome emerges, behavioural modification may prove an impactful means of cancer prevention.

**Supplementary Information:**

The online version contains supplementary material available at 10.1186/s13053-024-00280-w.

## Introduction

Lynch syndrome (LS) is a cancer predisposition syndrome caused by constitutional pathogenic variants in the genes *MLH1*, *MSH2*, *MSH6*, *PMS2* or *EPCAM* [1, 2]. It is the most common cause of hereditary colorectal and endometrial cancer, accounting for roughly 3% of each malignancy [3, 4]. LS is also associated with an increased risk of ovarian cancer, gastric cancer, small bowel carcinoma, pancreatic carcinoma, urothelial carcinoma, sebaceous carcinoma, and prostate cancer, among others [5].The lifetime risk of cancer varies widely among affected individuals with LS, based on factors such as genetic variant, family history, age, sex, and lifestyle [6]. This leads to both a challenge in risk stratification and a potential opportunity in cancer prevention.

Current methods of colorectal cancer prevention in LS include regular colonoscopies, for removal of pre-cancer polyps in addition to surveillance, and regular aspirin for chemoprevention [7]. In women, risk-reducing hysterectomy, with or without bilateral salpingo-oophorectomy depending on the gene-specific risk [7], is recommended once childbearing is complete to reduce risk of endometrial cancer [8]. These interventions while successful in reducing cancer risk [9], and increasing overall survival in the case of colonoscopy [10], generate anxiety and have morbidity for patients. A residual risk of malignancy remains [11], and people living with LS may benefit from guidance on what individual actions they can take to help mitigate their cancer risk [12, 13]. Moreover, there are no evidence-based recommendations for surveillance or prevention of other LS-associated cancers, which are shown to account for most of the mortality associated with the condition [14].

The most recent recommendations from the World Cancer Research Fund (WCRF) Continuous Update Project Expert Report advise reducing excess body weight, increasing physical activity, and minimising alcohol and tobacco consumption to reduce risk of colorectal and endometrial cancer [15]. Despite extensive data in the general population, evidence is only recently emerging on the role of these factors in the context of LS [16]. There have been several high-quality studies that show an association between several risk factors, such as obesity, lack of physical activity, and alcohol intake, and colorectal cancer in LS [17–27]. Data for endometrial cancer are sparce [28], and there is even less in other Lynch-associated cancers. Although studies have looked at the relationship between individual risk factors and relationship with cancer risk, there is a lack of data pertaining to potential modifiable risk profile and lifestyle behaviours of those with LS.

We have conducted an international, cross-sectional survey of modifiable risk factors in people with LS. Through this, we aim to characterise the burden of modifiable risk factors in patients with LS and how this compares to international guidelines for cancer prevention.

## Methods

### Questionnaire development

A questionnaire was developed with patient and public involvement (PPI) via partnership with Lynch syndrome Ireland (lynchsyndromeireland.com), a support and advocacy group for Irish people and families affected by LS. An initial draft survey was an abbreviated version of the Colon Cancer Family Registry [18] baseline questionnaire on epidemiology, risk factors and family history. Modifiable risk factors were chosen based on evidence-based risk factors for colorectal and endometrial cancer [29, 30], and consultation with international guidelines for cancer prevention including the WCRF [15, 31]. The survey was piloted and co-produced through a focus group of three Irish people living with LS. Feedback on the content and modality of this questionnaire was incorporated. Meetings were held with two patient representatives from LS Ireland (P.F and R. H.) throughout this project to guide research direction and methodology. The final questionnaire included 95 multiple-choice or free-text questions in 6 sections: demographic information (age, country of residence, sex assigned at birth), personal history of cancer, family history of cancer, modifiable risk factors for cancer, cancer surveillance and aspirin use, and female hormonal risk factors (Supplementary table S1). Ethical approval was granted from St James’s Hospital/Tallaght University Hospital Research Ethics Committee (ID 2128).

### Data collection

Anonymous data was collected via a secure online platform (forms.office.com) from March 28th to April 28th, 2023. Dissemination of the survey was carried out in partnership with LS Ireland. Participants were identified by invitation via a private group limited to those with LS in Ireland, alongside similar organisations in the UK, USA, and Finland. Social media was also used to invite people to take part.

### Statistical analysis

Survey responses with more than 50% missing data were removed from data analysis. Numerical data was presented as a mean and standard deviation or a median with an interquartile range depending on the distribution. For categorical data, proportions and frequencies were given. Differences in categorical survey responses between different subgroups (e.g., geographical region, cancer history) were compared using a Chi-squared test. To benchmark the modifiable risk profile of people with LS, results were compared against the AICR/WCRF guidelines for cancer prevention [15]. This includes 9 evidence-based lifestyle recommendations for cancer prevention, agnostic of tumour type, which apply to both the general population and cancer survivors. A total score of adherence to WCRF guidelines was calculated using methods described previously [32], which has been independently associated with cancer risk and mortality [33]. For the adherence score, we excluded the recommendation on breastfeeding, as it only applied to a subset of participants that had children. We also excluded the recommendation against supplement use, as there is no evidence that supplementation increases the risk of Lynch associated cancers. This contrasts to the association between high dose beta-carotene supplements and lung cancer [34], while there is some evidence that calcium supplementation may be protective against Lynch associated colorectal cancer [35]. Thus, the score was a sum of assigning either 1 (full adherence), 0.5 (partial adherence) or 0 (non-adherence) for each of the 7 remaining recommendations, with a score of 7 indicating full adherence. Further detail on scoring can be found in the supplementary table S2. The median adherence scores in different subgroups were compared using a Mann-Whitney *U* test. All statistical analysis was conducted on STATA 18.

## Results

### Baseline demographics

There were 156 respondents to the survey after excluding 1 entry due to < 50% missing data. The majority (88%) of were women, with a median age of 51 years. Baseline demographics are shown in Table 1. All participants had a diagnosis of LS, including 54 with a pathogenic variant in *MSH2* (34%), 39 in *MSH6* (25%), 38 in *MLH1* (24%), 17 in *PMS2* (11%), and 4 in *EPCAM* (3%). In total, 54% (84/156) had a previous cancer diagnosis, mostly colorectal or endometrial cancer, and 28% have been diagnosed with more than one primary cancer. Details of the personal and family history of the participants can be found in the supplementary material (Table S3).

**Table 1:** Baseline demographics of 156 survey participants. *Denotes participants from Germany, India, Singapore, Egypt, New Zealand and Italy

Sex	*N* (%)
Male	23 (12%)
Female	133 (88%)
**Median age**	51 (IQR 39–58)
**Country of residence**	
UK	62 (39%)
USA	50 (32%)
Ireland	24 (15%)
Finland	6 (4%)
Canada	5 (3%)
Australia	2 (1%)
Other*	7 (5%)
**Previous cancer diagnosis**	
Yes	84 (54%)
No	72 (46%)
**MMR PV detected**	
*MSH2*	54 (34%)
*MSH6*	39 (25%)
*MLH1*	38 (24%)
*PMS2*	17 (11%)
*EPCAM*	4 (3%)
Unknown / Not tested	4 (3%)
**Method of recruitment**	
Lynch syndrome private group	79 (50%)
Social media	61 (39%)
Family member	16 (11%)

### Prevention-related health behaviours

Among participants, 46% (72/156) reported regular aspirin use (Table 2). Regular aspirin use for chemoprevention was more frequent in those with a previous diagnosis of cancer (52% vs. 36%, *Χ*^*2*^ = 4.15, *p* = 0.04) and residents in Europe compared to North America (50% vs. 33%, *Χ*^*2*^ = 4.01, *p* = 0.04), but there was no significant difference in those were older than 50 years old, compared to younger participants (48% vs. 44%, *Χ*^*2*^ = 0.24, *p* = 0.62). Extra-colonic cancer surveillance was more frequent among participants residing in North America compared to European countries (65% vs. 41%, *Χ*^2^ = 8.1, *p* < 0.001).

**Table 2:** Behaviours surrounding cancer prevention among participants. *4 participants could not remember their aspirin dose

Aspirin usage	*N* (%)
Yes	72 (46%)
No	84 (54%)
Aspirin dose *	
75 or 81 mg daily	30 (19%)
300 mg or 325 mg daily	21 (13%)
150 mg daily	12 (8%)
600 mg daily	2 (1%)
75 or 81 mg three times a week	2 (1%)
150 mg three times a week	1 (1%)
**Frequency of colonoscopies**	
Yearly	63 (39%)
Every 2 years	73 (46%)
Every 3 years	3 (2%)
18 monthly	1 (1%)
Every 5 years	1 (1%)
Other	1 (1%)
No regular colonoscopy	14 (8%)
**Risk reducing surgery**	
Hysterectomy and salpingo-oophorectomy	56 (36%)
No surgery	102 (64%)
**Surveillance for other LS cancers**	
Upper GI endoscopy	43 (27%)
Endometrial surveillance (hysteroscopy and/or endometrial sampling)	10 (6%)
TVUS	14 (9%)
Cystoscopy or urine cytology	14 (9%)
Skin examination	8 (5%)

### Modifiable risk factors

The mean body mass index (BMI) of this cohort was 26.7 kg/m2 (Table 3). The participants reported mean of 90 min of moderate, or high-intensity physical activity weekly. Median weekly alcohol intake was 30 g of ethanol. There were 4 (3%) participants that reported current smoking, and 36% (53/156) were ex-smokers. Overall, 80% (126/156) of participants reported red meat intake, with a median weekly serving of 2. A smaller majority (65%) reported regular intake of processed meat, with median weekly servings of 1. The median daily intake of fruit and vegetables was 2 servings for both. 69% (108/159) reported daily dietary supplement usage.

**Table 3:** Modifiable risk factors among LS participants

BMI (kg/m2)	*N* (%)
18–25	66 (44%)
25–30	56 (35%)
30+	34 (21%)
Mean BMI (SD)	26.7 (6.1)
**Comorbidities**	N (%)
T2DM	9 (6%)
HTN	24 (15%)
Hyperlipidaemia	27 (17%)
Coeliac	3 (2%)
**Physical activity**	
Mean step count	9146 /day
Mean weekly minutes of physical activity	90
**Alcohol consumption**	N (%)
Current	111 (71%)
Former or never	45 (29%)
Median weekly units of alcohol	3
**Smoking status**	N (%)
Never	99 (63%)
Former	53 (36%)
Current	4 (3%)
Median pack years	5

**Fig. 1:**
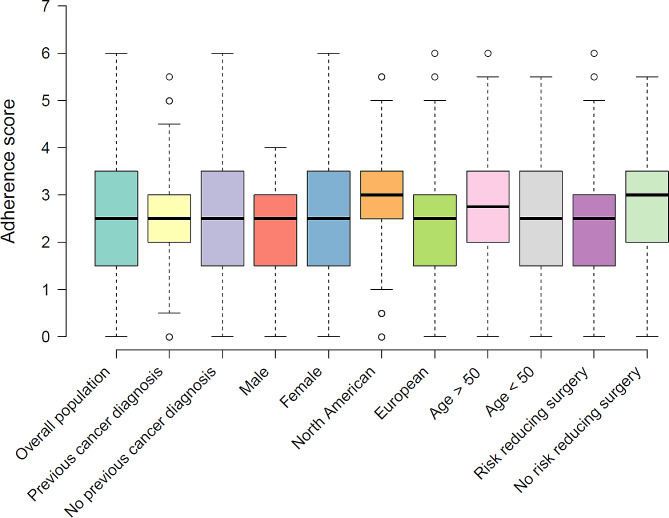
Boxplot of adherence scores to WCRF lifestyle recommendations in the overall population and subgroups Legend: Adherence scores were calculated by sum of assigning either 1 (full adherence), 0.5 (partial adherence) or 0 (non-adherence) for each of the 7 recommendations (excluding breastfeeding and supplement usage), with a score of 7 indicating full adherence. Centre lines show the medians; box limits indicate the 25th and 75th percentiles; whiskers extend 1.5 times the interquartile range from the 25th and 75th percentiles, outliers are represented by dots

To benchmark health and lifestyle behaviours against international guidelines for cancer prevention, we compared prevalence of modifiable risk factors against WCRF recommendations (Table 4). The median adherence score [32] among all participants was 2.5, out of a maximum of 7 (Fig. 1). There was no significant difference in median adherence score between males and females (2.5 vs. 2.5, *U* = 1375, *p* = 0.31), comparing cancer survivors with people with no cancer history (2.5 vs. 2.5, *U* = 2935, *p* = 0.75), between participants aged less than or greater than 50 years (2.75 vs. 2.5, *U* = 2733, *p* = 0.27), and between women who had underwent risk reducing surgery versus those that did not (2.5 vs. 3, *U* = 1710, *p* = 0.11). Residents of North America had a slightly higher adherence score compared to those residing in Europe (3 vs. 2.5, U = 2078, *p* = 0.024).

**Table 4:** World Cancer Research Foundation (WCRF) guidelines for cancer prevention. *Data was not collected regarding duration of breastfeeding. The figure of 73% denotes that 70 of 96 women reported breastfeeding, regardless of duration

AICR/WCRF recommendations	Details	Adherence
*Be a healthy weight*	Keep your weight as low as you can within the healthy range throughout life (BMI of 18.5–24.9)	65/156 (41%)
*Be physically active*	Be at least moderately physically active and follow or exceed national guidelines (i.e., 150 min of moderate intensity activity and 2 days of strength training)	25/156 (16%)
*Eat wholegrains, vegetables, fruit, and beans*	Eat a diet high in all types of plant foods including at least five portions or servings (at least 400 g or 15oz in total) of a variety of non-starchy vegetables and fruit every day	13/156 (8%)
*Limit fast foods*	Limit consumption of processed foods high in fat, starches, or sugars – including ‘fast foods’; many pre-prepared dishes, snacks, bakery foods and desserts; and confectionery (candy)	75/156 (48%)
*Limit red and processed meat*	If you eat red meat, limit consumption to no more than about three portions per week. Three portions are equivalent to about 350–500 g (about 12–18oz) cooked weight. Consume very little, if any, processed meat.	46/156 (29%)
*Limit sugar sweetened drinks*	Do not consume sugar sweetened drinks	77/156 (49%)
*Limit alcohol consumption*	For cancer prevention, it’s best not to drink alcohol	47/156 (40%)
*Do not use supplements for cancer prevention*	High-dose dietary supplements are not recommended for cancer prevention – aim to meet nutritional needs through diet alone	48/156 (31%)
*For mothers: breastfeed your baby if you can*	The Expert Panel endorses the advice of the World Health Organization, which recommends infants are exclusively breastfed for six months, and then for up to two years or beyond alongside appropriate complementary foods.	70/96 (73%)*

### Female hormonal risk factors

Female-specific risk factors for endometrial cancer are displayed in Table 5. Most women who had children breastfed (73%). Overall, 41% (56/134) of women have undergone a risk-reducing hysterectomy. Of the 99 post-menopausal women, 32% (*n* = 32) reported postmenopausal hormonal replacement therapy (HRT) with a median duration of use of 4.5 years. The most common preparation was oestrogen-only HRT (53%), and the most frequent method of delivery was an oral tablet (47%). The majority reported hormonal contraceptive use > 1 year, with a median duration of 10 years.

**Table 5:** Female specific risk factors for endometrial cancer in participants with Lynch syndrome. *7 could not recall the HRT preparation taken

Breastfeeding history	*N* (%)
Yes	70 (52%)
No	26 (19%)
Not applicable	38 (28%)
**Risk reducing surgery**	
Hysterectomy and salpingo-oophorectomy	56 (41%)
No surgery	78 (59%)
**Hormonal contraceptive use > 1year**	
Yes	107 (79%)
No	27 (20%)
Median duration of use	10 years
**Menopausal status**	
Pre-menopausal	35 (26%)
Post-menopausal	99 (74%)
Median age at menopause	46
**Post-menopausal hormone replacement** **therapy (HRT) use**	
Yes	32 (32%)
No	64 (68%)
Median duration of use	4.5 years
Hormonal HRT content *	
Oestrogen only	17 (53%)
Combined	7 (21%)
**Method of HRT delivery**	
Pill	15 (47%)
Patch	9 (28%)
Gel	7 (22%)

## Discussion

In this international cross-sectional survey, we have characterised the health and lifestyle behaviours among people living with LS. We show a substantial burden of modifiable risk factors in this high-risk subgroup. The WCRF guidelines on cancer prevention were chosen due to a strong evidence base and measurable targets for comparison. Several studies have associated adherence to WCRF guidelines with reduced risk and incidence of general and site specific cancers [36–39], as well as reduced all-cause and cancer specific mortality [40, 41]. Adherence scores in the general population vary based on the population sample, data collection methodology, and how the recommendations are operationalised into a score, but generally range from 3 to 4 [38, 42, 43], compared to 2.5 in this study. Applying this WCRF recommendations to LS specifically, a recent Dutch cross sectional study investigated determinants of adherence to WCRF recommendations on physical activity, red and processed meat intake, and body weight [44]. It found similar rates of adherence to recommendations on bodyweight (50%), and red and processed meat consumption (33%) to this study. Unlike this study, a higher proportion (78% compared to 16%) adhered to guidelines on physical activity. Similarly, a cancer history was not associated with increased adherence to lifestyle recommendations, but they found a relationship between age and adherence which was not seen here. However, that cross sectional study examined just 3 WCRF recommendations, compared to the 9 examined in this study. Furthermore, this was limited to the Dutch population compared to our worldwide sample.

We hypothesised that people living with LS, particularly with a cancer diagnosis, would have a low burden of modifiable risk factors and thus high rates of adherence to cancer prevention guidelines. This could be in part due to the inherited genetic aetiology driving their cancer diagnosis. We also hypothesized that a cancer diagnosis may facilitate behavioural change. This effect has been described in some studies in the general population [45, 46]. We found no association between a cancer diagnosis and adherence score to WCRF recommendations in LS in this sample. A recent prospective cohort study provided a detailed investigation into how a colorectal cancer diagnosis affects dietary and lifestyle habits in people with LS [47]. This longitudinal data from the GEOlynch cohort identified no difference in diet, body weight, or physical activity following a cancer diagnosis, but did find a reduction in smoking. This concordance in modifiable risk between cancer survivors and unaffected people with LS is consistent with this study.

This study demonstrates trends in chemoprevention and surveillance that differ between regions. We found that 46% report regular aspirin use, more common in Europeans compared to North Americans. Long term follow-up from the CAPP2 randomised trial showed that 600 mg of aspirin daily reduces risk of colorectal cancer in LS [17]. The CAPP3 trial is currently ongoing to determine the optimal dose for chemoprevention while limiting adverse effects, while the ideal start age and duration is also not clear [48]. Both European [7, 49] and USA-based guidelines [50] advise consideration of aspirin use to reduce colorectal cancer risk [7], but emphasise shared decision-making. This discrepancy may be explained by health-system and demographic factors that differ between the continents. The finding that aspirin use was more common in cancer survivors was of interest, as this group had a less potential lifetime benefit in terms of cancer prevention, and potentially higher risk of adverse effects. This may be explained by low dose aspirin use for cardiovascular indications in addition to chemoprevention, and more contact with secondary healthcare services. Although cancer survivors were generally older, we did not find an associated between age and aspirin usage. There was a higher prevalence of self-reported aspirin use among European (mainly UK) participants compared to those in North America. In addition to important healthcare system differences, the fact that CAPP2 and CAPP3 trials are led by the UK may also have influenced earlier adoption of aspirin for chemoprevention. This study also demonstrated more extra-colonic surveillance in respondents residing in the USA and Canada compared to Europe. This may be explained by differences in guidelines and therefore clinical practice. UK guidelines do not recommend gynaecological cancer surveillance [8, 49], or gastric cancer surveillance unless there is relevant family history, while US-based National Comprehensive Cancer Network (NCCN) guidelines recommend regular upper gastrointestinal endoscopy and the consideration of endometrial sampling [50].

We have described female-specific risk factors for endometrial cancer in LS patients. Historic data and research have focused on colorectal cancer, including the previous terminology of Hereditary Non-Polyposis Colorectal Cancer. There is clear need to improve awareness both amongst patients and providers about the impact of gynaecologic cancers for women with LS. A 2020 survey from the UK revealed wide variation in services and knowledge pertaining to LS among gynaecological oncologists [51], and this was also prominent theme in the PPI focus group in our study. The same UK survey reports a numerically higher prevalence of previous hysterectomy (64%) compared this cohort (41%). As the median age of participants was similar, perhaps this is due to the inclusion of non-UK residents in this cohort. The UK survey did not explore postmenopausal HRT, but we found it to be reported in roughly one-in-three post-menopausal women with LS, most (75%) of which have had a previous hysterectomy. Although there has been some progress in recent years in evidence-based recommendations of HRT in LS [52, 53], there is a pressing need for more data and robust guidelines to standardise clinical practice.

This study has several strengths. It is the most comprehensive and up-to-date cross-sectional study of modifiable risk factors among patients with LS with representation from diverse geographic regions. It provides an important context of health and lifestyle behaviours in those living with LS. This context provides opportunities to raise awareness of the role of lifestyle modifications in LS and other high penetrance cancer predisposition syndromes, potentially contributing to cancer prevention in high-risk groups. This context also can inform advocacy and lobbying efforts for support from healthcare services for this avenue of risk reduction. Another important strength is the emphasis of public and patient involvement. There was extensive input from patients advocates and people living with LS throughout this project. Patients had input into the planning, dissemination, and production of the questionnaire. In particular, the method of dissemination through patient networks, may prove a worthwhile model for research for people with hereditary cancer predisposition in the future.

The study has several implications for research and clinical practice. We have provided a comprehensive baseline characterisation of lifestyle risk in an international cohort of people with LS. Given that the median adherence score to recommendations is 2.5 out of 7, there is substantial scope for interventions that encourage behavioural change as an means of cancer prevention. Several studies show that the majority of colorectal cancer risk factors are applicable to those with LS [16, 54], but there is sparse data in endometrial cancer and other Lynch associated malignancies [16]. Regardless, the WCRF score has been associated with all-cause and site-specific cancer incidence and mortality in the general population, so behavioural interventions to increase adherence to these criteria may still have substantial benefit for those with LS. However, a systematic review of 4 randomised trials of behavioural intervention in people with genetic tumour syndromes showed mixed results [55]. One randomised trial included investigated the effect of providing WCRF health promotion materials in people with LS [56]. It found that although this information increased awareness and knowledge of the recommendations, it was not associated in increased adherence to these health behaviours. This highlights the complexity of achieving lasting changes to lifestyle in this population, and that awareness is not enough to effect lasting health behavioural change that could potentially modify cancer risk. The psychological burden of living with LS may contribute to this. Feelings of guilt, anxiety about results of surveillance tests, and the familial implications of a diagnosis may imperil the already difficult task of achieving lasting behavioural change [12]. Integration of specific psychological services into the LS multidisciplinary clinical team may not only improve wellbeing, but willingness to engage in healthy lifestyle behaviours. Financial barriers to healthy lifestyle habits are also relevant, which may be worsened by the well-described ‘financial toxicity’ of cancer treatment, if individuals LS already have a cancer diagnosis [57]. Financial supports designed to support health behavioural change could be worth exploring as a potentially cost-effective method cancer prevention.

Higher quality, prospective cohort studies are needed to better inform health and behavioural interventions in LS. This is particularly needed in non-colorectal Lynch-associated malignancies, where epidemiological data is lacking and surveillance and prevention lags behind colorectal cancer [8, 28]. Lastly, international guidelines on LS should increase the emphasis on lifestyle modification as a means of cancer risk reduction. This has been included, with a caveat acknowledging the uncertainty in the literature, in newest editions of several recent guidelines [8, 49]. As actionable evidence emerges in the coming years regarding modifiable risk factors in LS, strong guideline recommendations will be needed to influence clinical practice. Involvement of people with LS through PPI in development of these guidelines is needed to ensure these recommendations have a real-world impact. An example of this in action is the Lynch Choices project [58], where there has been successful input from LS patient representatives in co-designing tools for shared decision making on surveillance, prevention and lifestyle choices.

There are several limitations of this work. Firstly, the survey methodology is vulnerable to selection bias, which may impact the generalisability of the results. Participants were more likely to be female and older and reside in English speaking countries. Although 13 countries were represented, 7 of these countries just had one participant (Germany, India, Singapore, Egypt, New Zealand, and Italy) and the results are mostly applicable to residents of the United States, Ireland and the United Kingdom. This female predominance may reflect the gender balance of LS support groups, and has been seen in other LS studies using survey methodology [59]. People that choose to participate in research related to lifestyle risk may also be healthier than the typical individual with LS. As the data on modifiable risk factors was self-reported and not objectively quantified, some elements of lifestyle risk such as diet, weight, and physical activity may be over or under-reported. Data regarding duration of breastfeeding was not collected. As such, it was not possible to determine exact adherence to the WCRF recommendation of breastfeeding exclusively for 6 months and up to two years, and the figure of 73% adherence should be interpreted with this caveat in mind. Lastly, although we used an adherence score for the WCRF guidelines, very recent work has operationalised these guidelines into a standardised scoring system [60]. Using this standardised system would increase reproducibility of our findings but was not possible as it includes certain data points (e.g. percentage of calories from ultra-processed foods) that were not collected in this survey.

## Conclusion

In conclusion, there is a substantial burden of modifiable risk factors in this international survey of people living with LS. In a comparison with international guidelines for cancer prevention, there are clear targets for intervention that are present regardless of age, sex, or personal cancer history. Along with the emerging body of evidence of the role of modifiable risk factor in LS, this provides a strong rationale for lifestyle modification as an adjunctive means of cancer prevention in people living with an inherited cancer risk.

### Electronic supplementary material

Below is the link to the electronic supplementary material.

Supplementary Material 1

## Data Availability

The datasets used during the current study are available from the corresponding author on reasonable request.

## Abbreviations

WCRF: World Cancer Research Fund
LS: Lynch syndrome
BMI: body mass index
HRT: hormone replacement therapy
NCCN: national comprehensive cancer centre network
