# Supplementary material for: Modifiable risk factors for cancer among people with lynch syndrome: an international, cross-sectional survey
Source: Hered Cancer Clin Pract. 2024 Jun 14;22:10. doi: 10.1186/s13053-024-00280-w (PMC11177364; doi:10.1186/s13053-024-00280-w)
Supplement: Supplementary file 1 — Supplementary Material 1 [file 13053_2024_280_MOESM1_ESM.docx]

**Table S1**: Questions in survey. This is excluding the first section and first 6 questions that constituted the consent form.

| Number | Question text |
| --- | --- |
| 1 | What age are you? |
| 2 | What is your gender? |
| 3 | What is your country of residence? |
| 4 | Have you been diagnosed with Lynch syndrome? |
| 5 | Have you had genetic testing for Lynch syndrome? |
| 6 | If so, what mutated gene was found? |
| 7 | Have you ever been diagnosed with cancer? |
| 8 | Have you been diagnosed with more than one type of cancer? |
| 9 | What type of cancer were you diagnosed with? |
| 10 | What age were you at diagnosis? |
| 11 | What was the first type of cancer that you were diagnosed with? |
| 12 | What age were you at diagnosis?2 |
| 13 | What was the second kind of cancer that you were diagnosed with? |
| 14 | What age were you at diagnosis?3 |
| 15 | Have you been diagnosed with more than two cancers? |
| 16 | What was the third kind of cancer that you were diagnosed with? |
| 17 | What age were you at diagnosis?4 |
| 18 | Do you have a family history of cancer? |
| 19 | Do you have a first degree relative (i.e. brother, sister, parent, child) who has been diagnosed with one of the following cancers? Please select all that apply. |
| 20 | Do you have multiple first degree relatives (i.e. brother, sister, parent, child) diagnosed with the same type of one of the above cancers? |
| 21 | Please state which kind(s) of cancer and which first degree relatives (i.e. brother, sister, parent, child) have been affected. |
| 22 | Do you have a second degree relative (i.e. uncle, aunt, grandparent, niece, nephew, half-sibling) with a diagnosis of the following cancers? Please select all that apply |
| 23 | Do you have multiple second degree relatives (i.e aunt, uncle, grandparent, niece, nephew) on the same side of the family diagnosed with the same type of one of the above cancers? |
| 24 | Please state which kind of cancer and which relatives (i.e aunt, uncle, grandparent, niece, nephew) have been affected. |
| 25 | What weight are you? |
| 26 | What height are you? |
| 27 | What level of activity is your job? |
| 28 | Do you track your daily step count? |
| 29 | What is your average daily step count? |
| 30 | Do you regularly exercise? |
| 31 | What is the most frequent type of exercise that you do? |
| 32 | How many days, on average per week, do you do this activity? |
| 33 | How many minutes does an average session of this activity last for? |
| 34 | Do you do any other types of exercise regularly? |
| 35 | What is the next most frequent type of exercise that you do? |
| 36 | How many days, on average per week, do you do this activity?2 |
| 37 | How many minutes does an average session of this activity last for? 2 |
| 38 | Do you do any other type of exercise regularly? |
| 39 | What is the next most frequent type of exercise that you do?2 |
| 40 | How many days, on average per week, do you do this exercise? |
| 41 | How many minutes does an average session of this exercise last for? |
| 42 | Do you drink alcohol? |
| 43 | How often, on average, do you drink alcohol? |
| 44 | How many units of alcohol would you drink in an average sitting? |
| 45 | Have you ever smoked tobacco? |
| 46 | Do you currently smoke? |
| 47 | How many cigarettes (or equivalents) do you smoke on an average day? |
| 48 | How many years have you smoked for? |
| 49 | How many cigarettes (or equivalents) did you smoke on an average day? |
| 50 | How many years did you smoke for? |
| 51 | Do you smoke e-cigarettes (vape)? |
| 52 | Do you eat red meat? |
| 53 | How many servings would you eat on an average week? |
| 54 | Do you eat ham, bacon, sausages or other deli meats? |
| 55 | How many servings would you eat in an average week? |
| 56 | How many servings of vegetables would you eat on an average day? |
| 57 | How many servings of fruit would you eat on an average day? |
| 58 | How often, on average, do you consume “fast foods” or other processed foods high in fat or sugars? |
| 59 | How often, on average, do you consume a sugar-sweetened drink? |
| 60 | Do you take any dietary supplements? |
| 61 | If so, what supplements do you take? |
| 62 | Have you ever been diagnosed with diabetes? |
| 63 | If so, what type? |
| 64 | Have you ever been diagnosed with coeliac disease? |
| 65 | Do you have high cholesterol? |
| 66 | Do you have high blood pressure? |
| 67 | Do you take aspirin or similar anti-inflammatory medications* regularly? |
| 68 | If you take aspirin, what daily dose do you take? |
| 69 | Do you undergo regular colonoscopies? |
| 70 | How often do you have a colonoscopy? |
| 71 | Have you underwent any risk reducing surgeries? |
| 72 | What risk-reducing surgery did you have? |
| 73 | Aside from colonoscopies, do you undergo any other regular screening tests for Lynch syndrome associated cancers? |
| 74 | What screening tests do you undergo? |
| 75 | Do you undergo any of the following regular cancer screening tests that are available to the general population? |
| 76 | Are you female? |
| 77 | If you have children, did you breastfeed? |
| 78 | Have you ever used hormonal contraceptives (e.g. 'the pill', implant, injections, or hormonal IUDs) for at least one year? |
| 79 | What kind of hormonal contraception did you/do you currently use? If multiple types, select whichever kind you used the longest. |
| 80 | In total, how many years did you take hormonal contraceptives for? |
| 81 | Have you had a menstrual period in the last 12 months? |
| 82 | How old were you when your periods stopped permanently? (i.e. entered menopause) |
| 83 | Have you ever used post-menopausal hormonal replacement therapy? |
| 84 | How many years did you take hormonal replacement therapy for? |
| 85 | What type of hormonal replacement therapy did you use? |
| 86 | What form was the hormonal replacement therapy that you used? |
| 87 | Would you like to be contacted regarding future studies like this? |
| 88 | Please provide your email address below |
| 89 | How did you hear about this survey? |

**Table S2:** scoring criteria for World Cancer Research Foundation (WCRF) recommendations.

| AICR/WCRF recommendations | Criteria | Score |
| --- | --- | --- |
| *Be a healthy weight* | BMI <25 | 1 |
|  | BMI 25-30 | 0.5 |
|  | BMI ≥30 | 0 |
| *Be physically active* | ≥150 minutes moderate or vigorous exercise per week AND 2 days of strength training | 1 |
|  | ≥150 minutes moderate or vigorous exercise per week OR 2 days of strength training | 0.5 |
|  | None of the above | 0 |
| *Eat wholegrains, vegetables, fruit, and beans* | ≥5 servings of fruit AND vegetables per day | 1 |
|  | ≥5 servings of fruit OR vegetables per day | 0.5 |
|  | None of the above | 0 |
| *Limit fast foods* | Consumption of foods high in saturated fat, salt, and sugar, including fast foods <1 serving per week | 1 |
|  | ≥1 serving per week | 0 |
| *Limit red and processed meat* | <3 portions red meat AND no processed meat | 1 |
|  | <3 portions red meat OR no processed meat | 0.5 |
|  | None of the above | 0 |
| *Limit sugar sweetened drinks* | Consumption of sugar-sweetened drinks <1 serving per week | 1 |
|  | Consumption of sugar-sweetened drinks ≥1 serving per week | 0 |
| *Limit alcohol consumption* | No regular alcohol intake (i.e <30g ethanol per week) | 1 |
|  | Alcohol intake (≥30g ethanol per week) | 0 |

**Table S3**: Personal and family history of cancer of participants with Lynch syndrome.

| **Personal history of cancer** | **N (%)** |
| --- | --- |
| No previous cancer diagnosis | 74 (47%) |
| 1 previous cancer diagnosis | 38 (24%) |
| 2 previous cancer diagnoses | 31 (20%) |
| 3 previous cancer diagnoses | 15 (9%) |
| **LS associated cancer diagnosed** |  |
| Colorectal | 45 (31%) |
| Endometrial | 33 (21%) |
| Ovarian | 7 (5%) |
| Urothelial | 6 (3%) |
| Prostate | 2 (1%) |
| Small bowel | 1 (1%) |
| **Family history of cancer** |  |
| **First degree relative** |  |
| Colorectal | 105 (67%) |
| Endometrial | 44 (28%) |
| Ovarian | 15 (10%) |
| Urothelial | 17 (10%) |
| Prostate | 16 (10%) |
| Gastric | 12 (8%) |
| Pancreatic | 14 (9%) |
| **Second degree relative** |  |
| Colorectal | 141 (90%) |
| Endometrial | 54 (34%) |
| Ovarian | 22 (14%) |
| Urothelial | 16 (10%) |
| Prostate | 14 (9%) |
| Gastric | 29 (18%) |
| Pancreatic | 18 (11%) |
